# Supplementary material for: Identifying associations of de novo noncoding variants with autism through integration of gene expression, sequence, and sex information
Source: Genome Biol. 2025 Jun 4;26:156. doi: 10.1186/s13059-025-03619-1 (PMC12139140; doi:10.1186/s13059-025-03619-1)
Supplement: Supplementary file 1 — Additional file 1. Supplementary text, Supplementary Figures S1-S10, Supplementary Tables S2 and S3. [file 13059_2025_3619_MOESM1_ESM.pdf]

Identifying associations of *de novo* noncoding variants with autism through integration of gene expression, sequence and sex information

Runjia Li[1] and Jason Ernst[1,2,3,4,5,6,7]
[1]Bioinformatics Interdepartmental Program, University of California, Los Angeles, Los Angeles, CA, 90095, USA.
[2]Department of Biological Chemistry, University of California, Los Angeles, Los Angeles, CA, 90095, USA.
[3]Eli and Edythe Broad Center of Regenerative Medicine and Stem Cell Research at University of California, Los Angeles, Los Angeles, CA, 90095, USA.
[4]Computer Science Department, University of California, Los Angeles, Los Angeles, CA, 90095 USA.
[5]Jonsson Comprehensive Cancer Center, University of California, Los Angeles, Los Angeles, CA, 90095, USA.
[6]Molecular Biology Institute, University of California, Los Angeles, Los Angeles, CA, 90095, USA.
[7]Department of Computational Medicine, University of California, Los Angeles, Los Angeles, CA, 90095, USA.

Correspondence: jason.ernst@ucla.edu (JE)

Contents:

- Supplementary Text
- Supplementary Figures S1-S10
- Supplementary Tables S2 and S3

Supplementary text

Local GC content differences between male and female samples

To assess the extent to which the observed signal might be associated with general differences between male and female samples, we repeated the local GC content analysis by comparing male siblings with female siblings, as well as comparing male probands with female probands. For both comparisons, no tissue reached the FDR-based significance threshold of 0.05. Between variants from male siblings (n=15,685) and female siblings (n=17,272) we note that 10 out of the 13 brain-related tissues are nominally significant ($p < 0.05$) (Figure S1f). Between variants from male probands (n=30,129) and female probands (n=4,496) one brain tissue was nominally significant ($p < 0.05$, Figure S1f) though we note the sample size of variants for female probands is limited in this analysis. These results provide suggestive evidence of differences between male siblings and female siblings though it did not reach the same level of significance as seen in the male proband-female sibling analysis. We emphasize that the SSC dataset only consists of samples from ASD families and thus siblings are not necessarily representative of unaffected individuals in the general population.

To further explore the potential sex differences among siblings, we restricted the analysis to siblings from families with a male proband (13,571 male sibling variants and 14,891 female sibling variants, Figure S1g). While no significant signals were found at an FDR threshold of 0.05, 9 out of the 13 brain-related tissues are nominally significant. We also compared variants from male probands with a female sibling (n=15,539) to male probands with a male sibling (n=13,680), observing no significant signal at an FDR threshold of 0.05 though two brain-related tissues were nominally significant ($p < 0.05$). These results suggest the signal originally observed between male proband-female siblings but not male proband-male siblings is not explained by only the difference between male probands that have male siblings and male

probands that have female siblings or the difference between male and female siblings that have male probands.

Clustering of the top neighborhoods identified by ENSAS

To examine the extent by which the top 28 neighborhoods characterize diverse genes and pathways, we clustered these neighborhoods using DBSCAN (Ester *et al.*, 1996) based on their variant compositions (Methods) and on each cluster repeated the GO enrichment analysis on the union of genes of all neighborhoods in the cluster, using the two different backgrounds described above. We observed that one cluster contained 24 neighborhoods while the remaining four clusters contained one neighborhood each. These four neighborhoods had on average between 462 and 587 variants overlapping with neighborhoods in the largest cluster, and on average a Jaccard index between 0.30 and 0.40 with neighborhoods in the largest cluster. The clusters showed consistency for the top enriched terms, with terms broadly related to synaptic transmission and cell junction being significant in all clusters (Figure S3). This suggests that the top neighborhoods identified by ENSAS implicate similar biological processes.

ENSAS proband-sibling local GC content differences stratified by sequencing phases

We note that whether the samples have matching or mismatching sequencing lanes is associated with their sequencing phases. Overall, the samples were sequenced across three primary phases, with phase 3 having two sub-phases. In total, 900 out of the 968 samples from pairs with matching sequencing lanes were sequenced in phases 2 and 3-1, while 590 out of the 606 samples from pairs with mismatching sequencing lanes were sequenced in phases 1, 3-2, and the pilot phase (Table S3). For each phase excluding the pilot phase, we performed proband vs. sibling Mann-Whitney U-tests separately for each neighborhood from the ENSAS analysis on the M-F upstream variants when restricting to the subset of variants from that phase

(Figure S4d-i). We observed that the overall signal was mainly concentrated in phase 2 (526 samples, Figure S4d) and to a lesser extent phase 3 (568 samples, when combining both 3-1 and 3-2 subphases, Figure S4d). The 28 brain-related neighborhoods significant in the full set of samples have median p-values of 8.2×10^{-5} and 4.6×10^{-4} in phases 2 and 3 respectively (Figure S4f, i). Overall, these analyses support there is no obvious explanation in terms of technical confounding. First, while the signal can be mainly attributed to the subset of lane-matching samples, those comparisons would be expected to be less susceptible to technical sequencing confounders and the association from the additional samples is in a consistent direction. Additionally, while phase 2 had the largest number of lane-matched samples and was most associated, the overall association was enhanced by considering samples from additional phases.

Supplementary figures

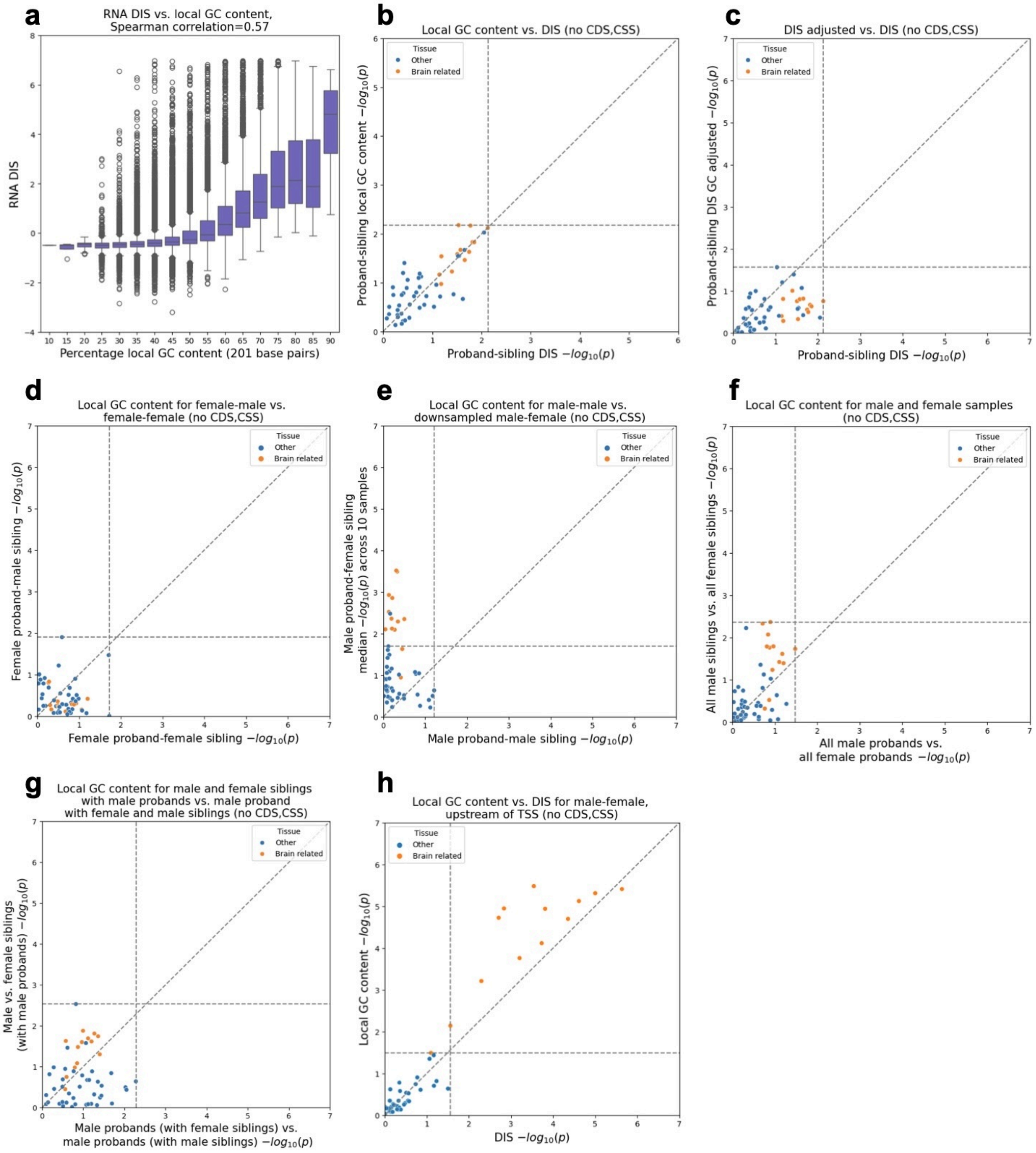

Figure S1 DIS vs. local GC content. Additional panels related to Fig. 1. **(a)** The y-axis corresponds to the RNA DIS score and the x-axis evenly sized local GC content intervals, each with size 10 (corresponding to 5%, with bin labeled as N representing N%-5% - N%). The boxes correspond to the quartiles, the lengths of whiskers correspond to 1.5x interquartile range and variants above/below the whiskers are defined as outliers. The reported Spearman correlations was computed between the RNA DIS and local GC content, across all variants. **(b-h)** Proband-sibling differences for DIS (combined) or local GC content of variants assigned to each

of the 53 GTEx tissue or cell types, with coding and canonical splice site (CSS) variants removed, are shown colored based on whether they are brain related or not. Both the x- and y-axis show -$\log_{10}$ p-values from one-sided Mann-Whitney U-tests. Horizontal and vertical dashed lines show p-values at FDR threshold of 0.05. Points greater than (but not on) these lines are significant after FDR correction. Diagonal dashed lines show the unit slope. **(b)** Proband-sibling differences for DIS vs. local GC content; **(c)** Proband-sibling differences for DIS (adjusted for local GC content) vs. proband-sibling differences for DIS. **(d)** Female proband-male sibling pair differences for local GC content vs. female proband-female sibling pair differences for local GC content. **(e)** Downsampled male proband-female sibling pair differences for local GC content, computed based on the median of ten subsets of 27,251 variants to match the number of male proband-male sibling variants, vs. male proband-male sibling pair differences for local GC content. **(f)** All male siblings-all female siblings differences vs. all male proband-all female proband differences for local GC content. **(g)** Male siblings (with male probands)-female siblings (with male probands) vs. male probands (with female siblings)-male probands (with male siblings) differences for local GC content. **(h)** male proband-female sibling pair differences in variants <100kbp upstream of nearest outermost TSS only for local GC content vs. for DIS.

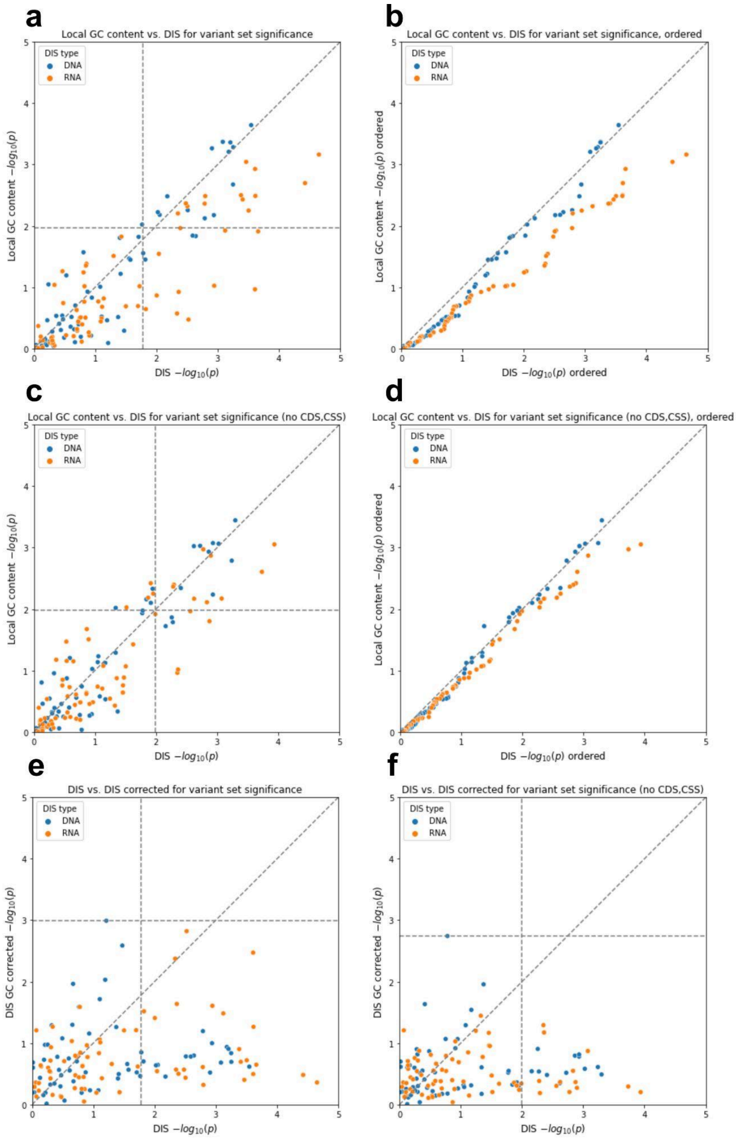

Figure S2 -$\log_{10}$ p-values from the genomic variant set analysis using the same testing procedure as described previously by Zhou *et al.*, 2019. All analyses excluded coding variants. Both the x- and y-axis show -$\log_{10}$ p-values from one-sided Mann-Whitney U-tests. Horizontal and vertical dashed lines show p-values at FDR threshold of 0.05. Points greater than (but not on) these lines are significant after FDR correction. Diagonal dashed lines show the unit slope. **(a)** proband-sibling differences for local GC content vs. proband-sibling differences for DIS with DNA and RNA-based tests colored separately **(b)** same as (a) but the scatter plot shows the *i*th most significant p-value for DIS against local GC content separately for the DNA and RNA-based tests for all possible values of *i*. **(c)** same as (a) but with CSS variants removed; **(d)** same as (b) but with CSS variants removed **(e)** proband-sibling differences for DIS (corrected for local GC content) vs. proband-sibling differences for uncorrected DIS; **(f)** same as (e) but with CSS variants removed.

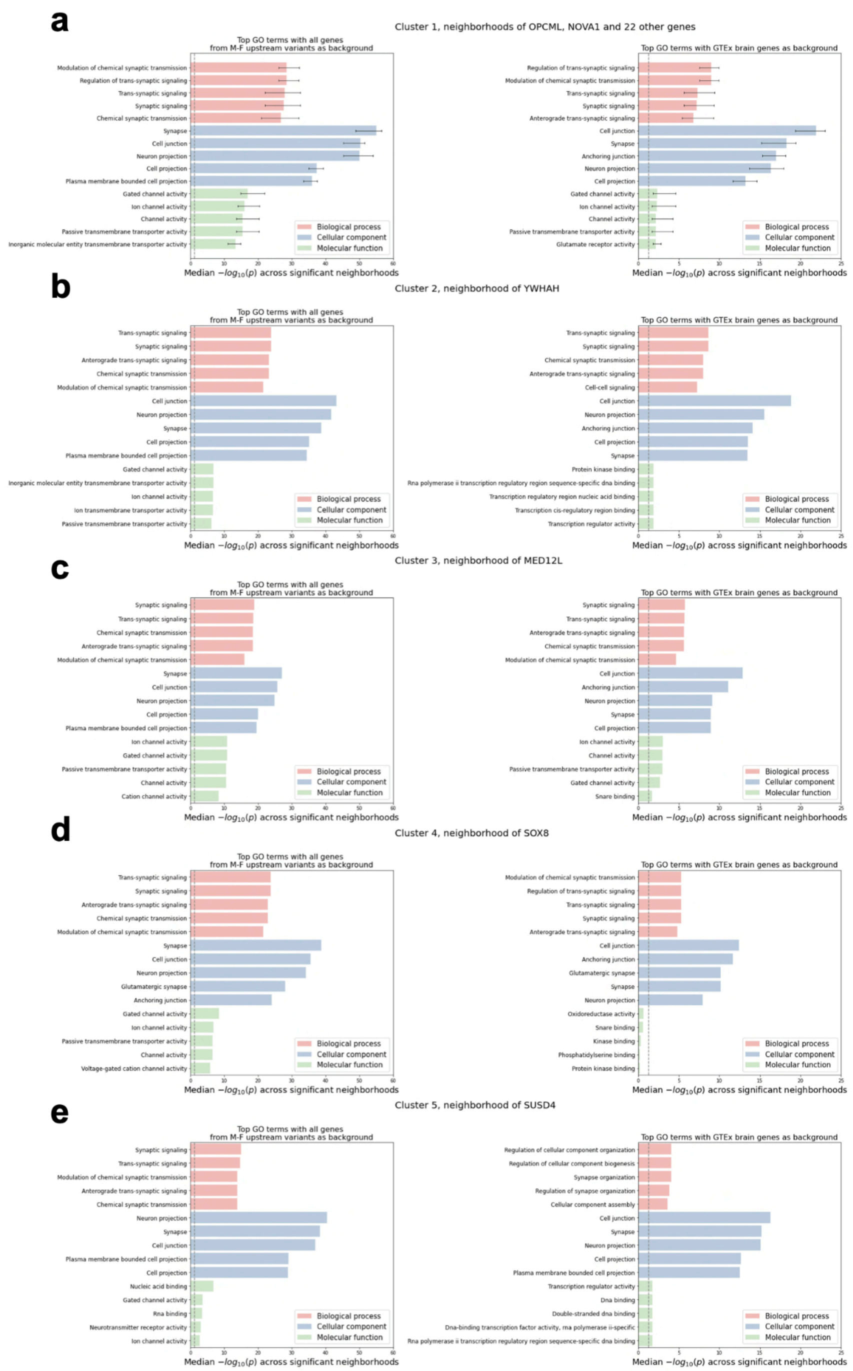

Figure S3 Top five enriched GO terms for each of biological processes, cellular component and molecular function terms for clusters of the top 28 significant neighborhoods in the M-F upstream variants ENSAS analysis. Shown are results using the union of all genes assigned to an M-F upstream variant as background (left) or the union of genes assigned to an M-F upstream variant that are also differentially expressed within any of the 13 brain-related GTEx tissues as background (right). x-axis shows Benjamini-Hochberg adjusted Fisher's exact -$\log_{10}$ p-values. Vertical dashed line shows p-value significance threshold of 0.05. **(a)** Median -$\log_{10}$ p-values across neighborhoods in the largest cluster with 24 neighborhoods. Error bars show

interquartile range across the neighborhoods. **(b-e)** P-values for the remaining clusters, each containing one neighborhood.

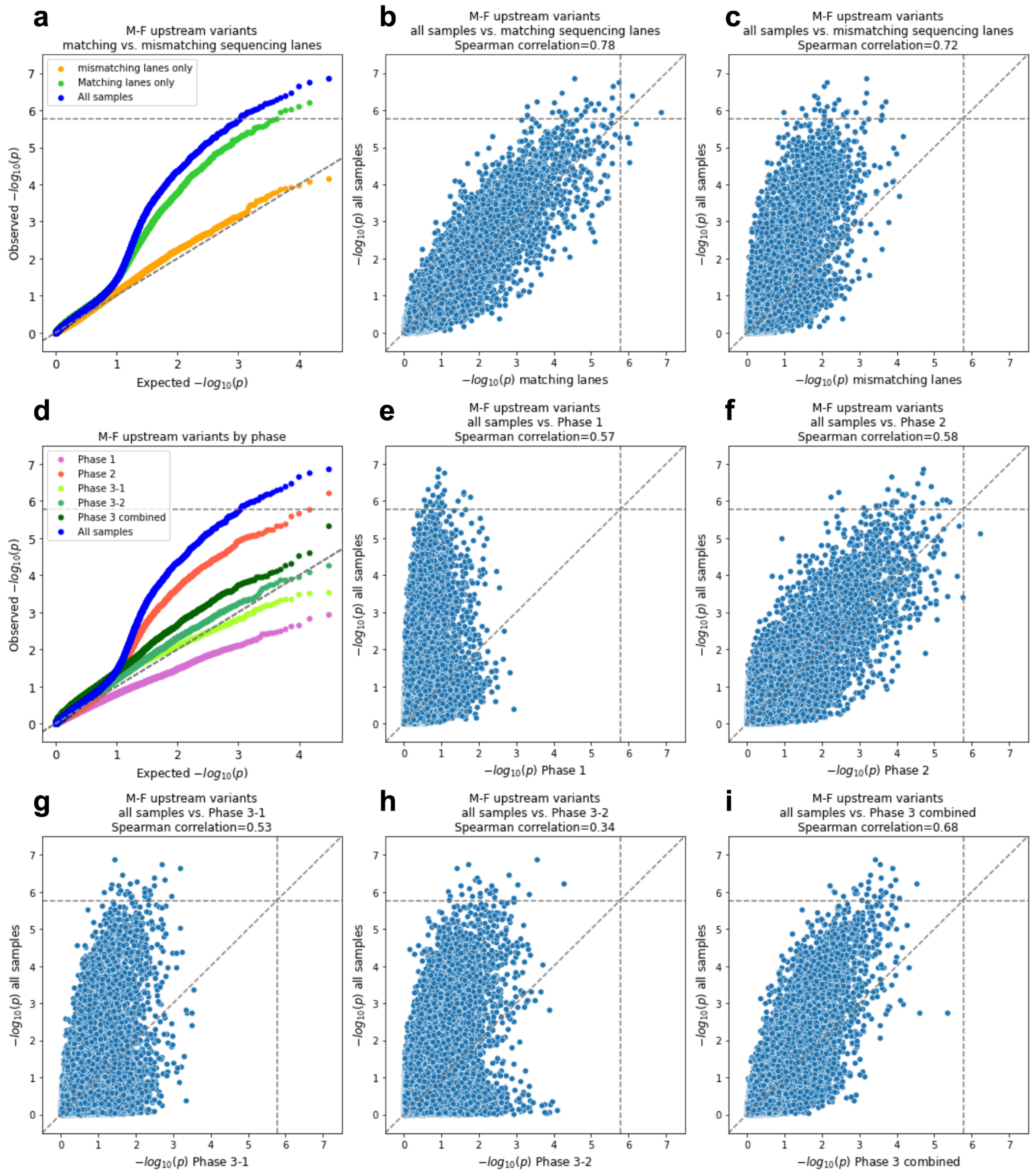

Figure S4 Comparison of local GC content Mann-Whitney U p-values for ENSAS on selected subsets of M-F upstream variants of all samples. Horizontal and vertical dashed lines show Bonferroni-based p-value multiple testing significance thresholds of 0.05 / n where n=29,820 is the number of neighborhoods. Diagonal dashed lines show the unit slope. **(a-c)** Samples with

matching sequencing lanes between probands and siblings (“matching lanes”), and samples with mismatching sequencing lanes between probands and siblings (“mismatching lanes”) are shown in a QQ-plot (a) and scatter plots (b-c). **(a)** QQ-plot for the neighborhood local GC content p-values of three sample groups. **(b)** Neighborhood p-values for all samples vs. samples with matching sequencing lanes. **(c)** Neighborhood p-values for all samples vs. samples with mismatching sequencing lanes. **(d-i)** Samples from sequencing phases 1, 2, 3 (with two subphases, 3-1 and 3-2) are shown in a QQ-plot (d) and scatter plots (e-i). **(d)** QQ-plots for the neighborhood local GC content p-values of each of the phases. **(e-i)** Neighborhood p-values for all samples vs. samples in each of the phases.

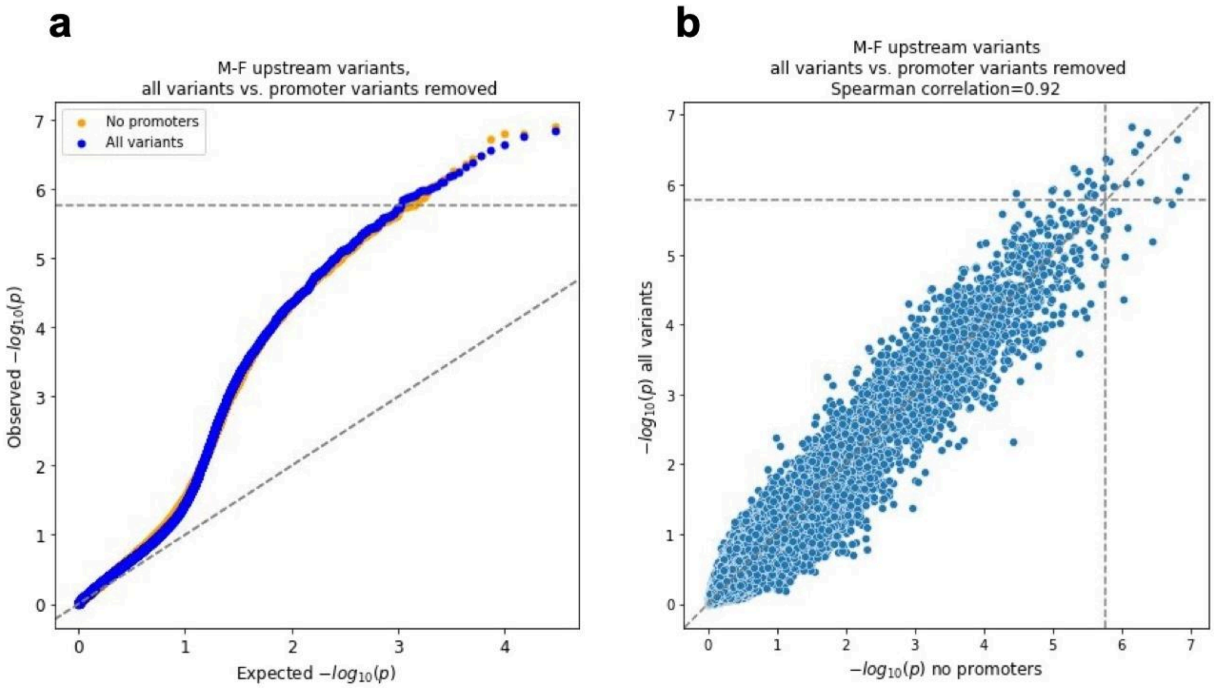

Figure S5 Comparison of local GC content neighborhood p-values from ENSAS on all M-F upstream variants vs. those excluding promoter variants. Horizontal and vertical dashed lines show Bonferroni-based p-value multiple testing significance thresholds of 0.05 / n where n=29,820 is the number of neighborhoods. Diagonal dashed lines show the unit slope. **(a)** QQ-plot of neighborhood p-values before and after removing promoter variants. **(b)** Neighborhood p-values for before vs. after removing promoter variants.

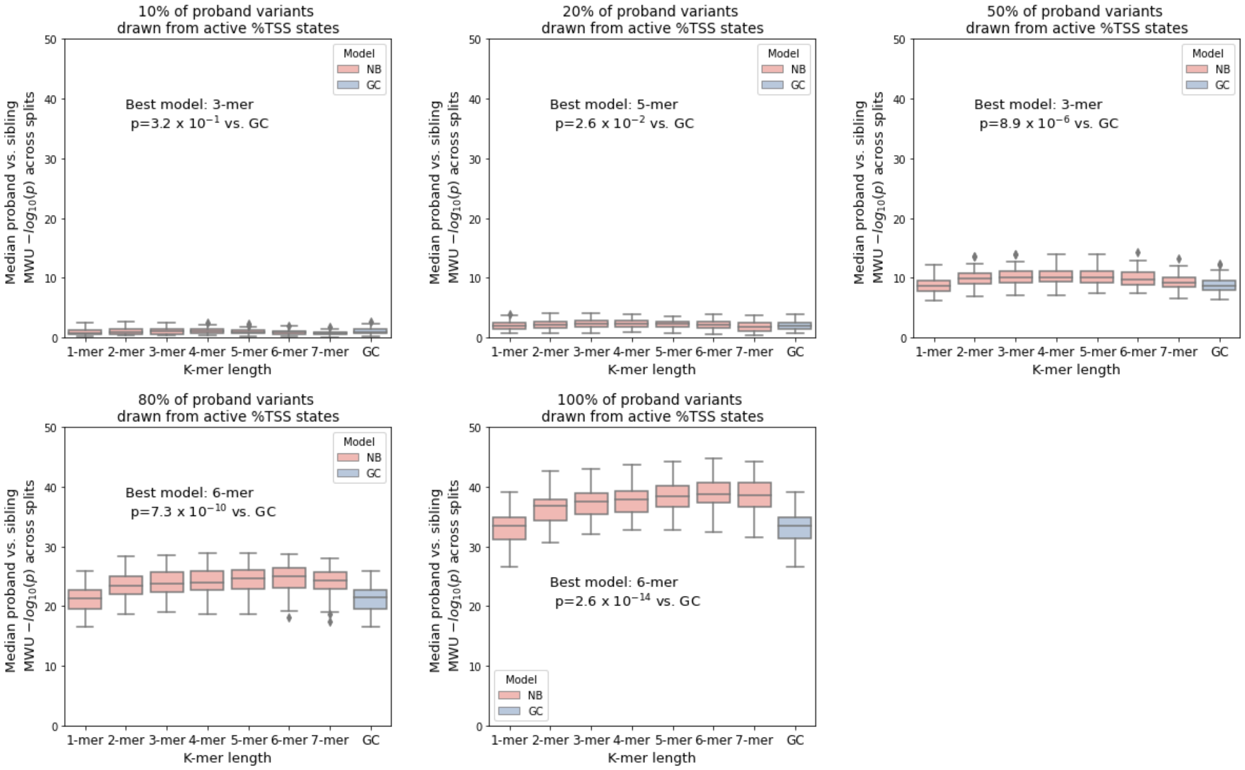

Figure S6 Median Naive Bayes (NB) model and local GC content (GC) p-values from ENSAS for proband vs. sibling variants across 50 random train-test splits on simulated datasets. Each subfigure represents 50 simulated datasets where the indicated portion of proband variants was drawn from active TSS-associated chromatin states (TssA, TssFlnk, TssFlnkU and TssFlnkD, Methods). Boxes show medians and interquartile ranges across 50 simulated datasets. Points above or below 1.5x interquartile range are drawn as outliers. Annotated text shows the best-performing k-mer model in terms of median performance across simulations and the one-sided Mann-Whitney U p-values of its performance vs. local GC content.

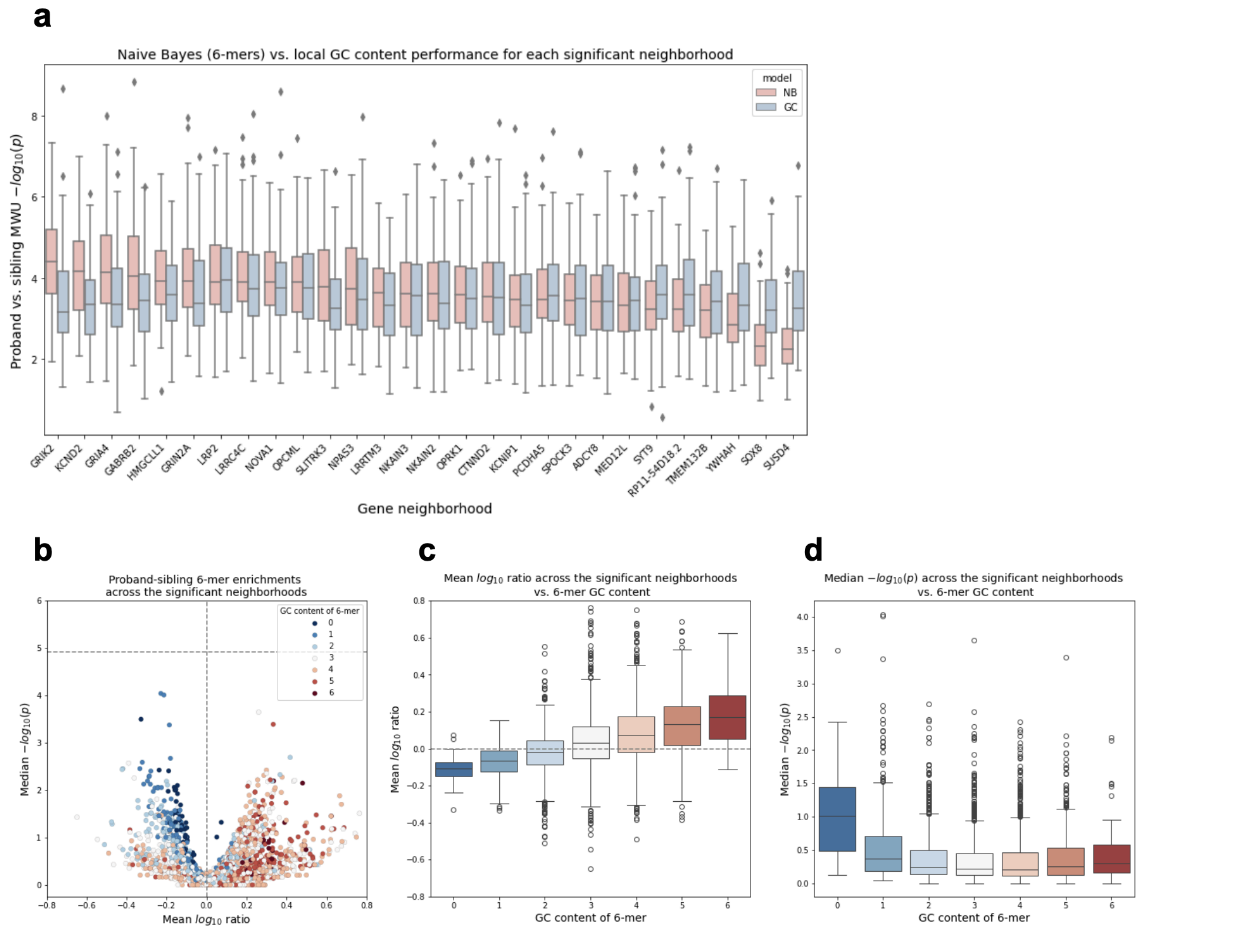

Figure S7 K-mer-based analysis for each of the 28 significant neighborhoods. **(a)** 6-mer Naive Bayes model (NB) and local GC content (GC) p-values from ENSAS across 100 random train-test splits for each of the 28 significant neighborhoods. Boxes show median and interquartile ranges. Points above or below 1.5x interquartile range are drawn as outliers. Each neighborhood is labeled by its central gene. The neighborhoods are sorted by their median Naive Bayes p-values. **(b)** Proband-sibling 6-mer enrichments across the 28 significant neighborhoods. Each point represents a unique 6-mer, colored based on its GC content. The x-axis is log_{10}((6-mer count in proband variants / 6-mer count in sibling variants) / (number of proband variants / number of sibling variants)) with the mean taken over 28 neighborhoods and positive values indicating preference for proband variants. The y-axis is the two-sided binomial p-value of the 6-mer's occurrence count in proband variants vs. sibling variants with the median taken over 28 neighborhoods. Horizontal dashed line shows Bonferroni-based p-value multiple testing significance threshold of 0.05 / n where $n=4^6$ is the total number of 6-mers. **(c)** Distributions of the mean log_{10} ratios (same as the x-axis in (b)) for 6-mers with different GC contents. **(d)** Distributions of the median binomial p-values (same as the y-axis in (b), but displayed on a different scale) for 6-mers with different GC contents. For both (c) and (d) boxes show median and interquartile ranges. Points above or below 1.5x interquartile range are drawn as outliers.

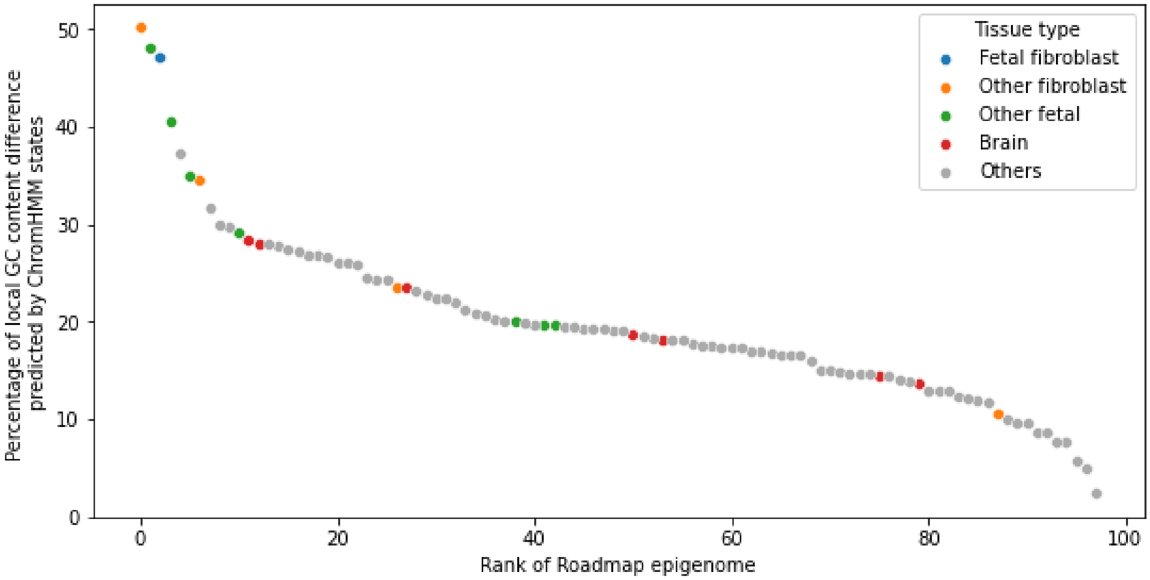

Figure S8 Percentage of differences in local GC content between proband and sibling variants predicted by the chromatin state annotations of the variants, ranked for each of the 98 epigenomes from Roadmap Epigenomics with annotations available based on the 18-state model. Selected subset groups of epigenomes are colored as follows: Blue: fetal fibroblast tissues; Orange: non-fetal fibroblast tissues; Green: non-fibroblast fetal tissues; Red: brain tissues; Grey: all other tissues.

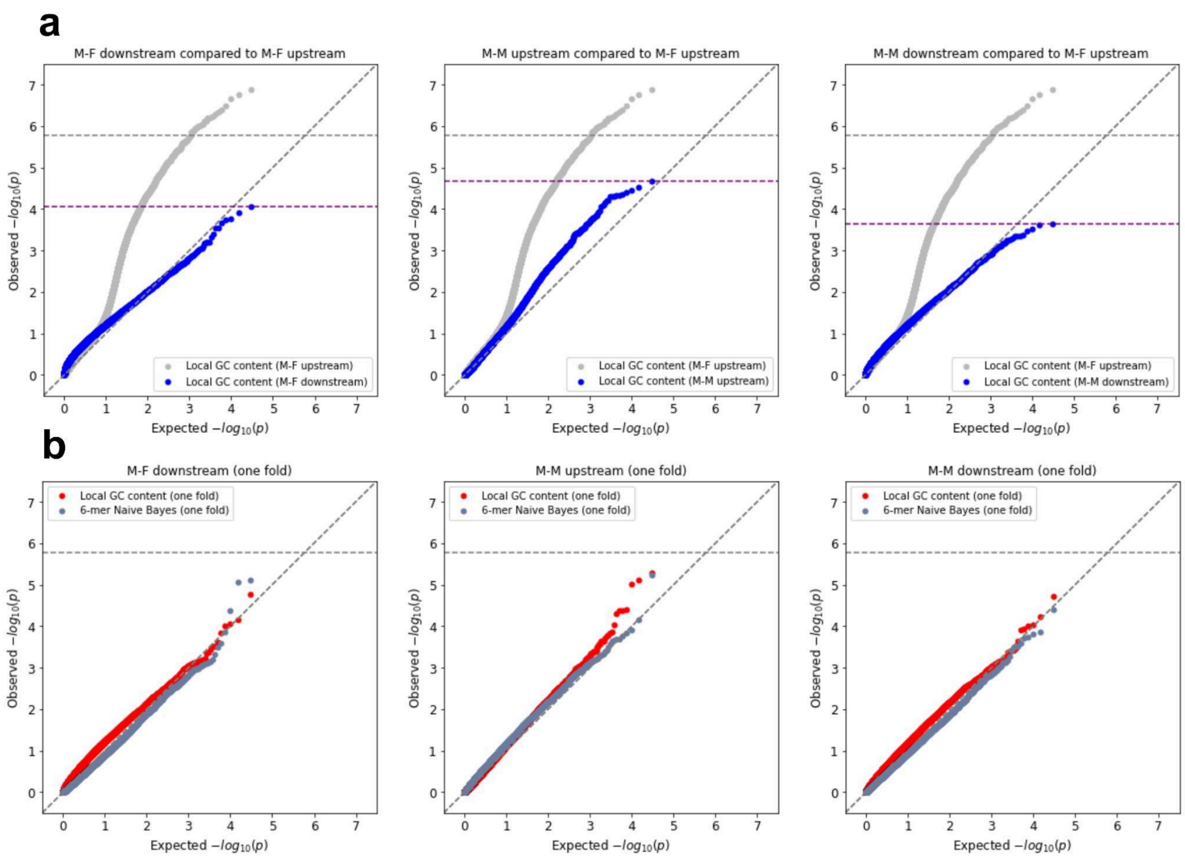

Figure S9 QQ-plots for Mann-Whitney U p-values from ENSAS for M-F downstream (left), M-M upstream (middle), and M-M downstream variants (right). Grey horizontal dashed line shows Bonferroni-based p-value multiple testing significance threshold of 0.05 / n where n=29,820 is the number of neighborhoods. Purple horizontal dashed lines show permutation-based multiple testing threshold at an FDR of 0.05, with points greater than (but not on) these lines significant after correction. Diagonal dashed lines show the unit slope. **(a)** P-values for local GC content using all variants in the neighborhood are shown in blue. P-values for the SSC M-F upstream analysis are also displayed in grey for comparison. **(b)** P-values for local GC content in red and 6-mer Naive Bayes model in grey on the testing fold for each neighborhood are shown.

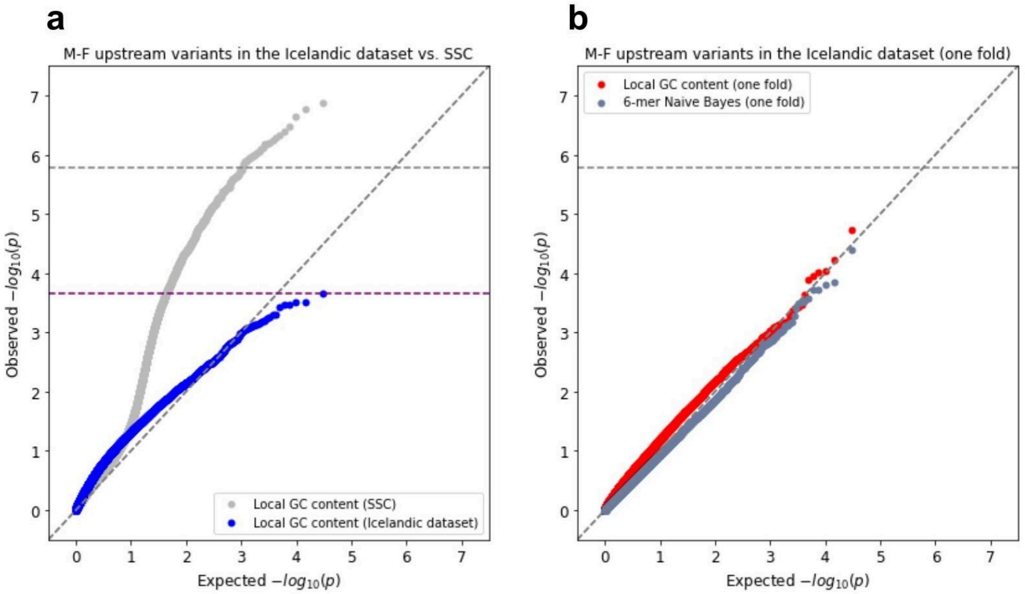

Figure S10 QQ-plot of Mann-Whitney U p-values from ENSAS for all M-F upstream variants in the Icelandic dataset from Halldorsson *et al.*, 2019. Grey horizontal dashed line shows the Bonferroni-based p-value multiple testing significance threshold of 0.05 / *n* where *n*=29,820 is the number of neighborhoods. Purple horizontal dashed line shows permutation-based multiple testing threshold at an FDR of 0.05 (Methods), with points greater than (but not on) these lines significant after correction. Diagonal dashed line shows the unit slope. **(a)** P-values for local GC content using all variants in the neighborhood are shown in blue. P-values for the SSC M-F upstream analysis are also displayed in grey for comparison. **(b)** P-values for local GC content in red and 6-mer Naive Bayes model in grey on the testing fold for each neighborhood are shown.

Supplementary tables

Table S2 Proband vs. sibling differences in the number of *de novo* coding variants or protein-truncating variants (PTVs). Two-sided binomial p-values are shown. Paternal age adjustments were performed following Werling *et al.*, 2018 and An *et al.*, 2018.

**Probands vs. siblings**	**Variant type**	**No adjustment**		**Adjusted for paternal age**	
		**Fold enrichment**	**P-value**	**Fold enrichment**	**P-value**
**All probands vs. all siblings**	**Coding variants**	1.01	0.25	1.01	0.20
	**PTVs**	1.20	6.3×10^{-4}	1.21	5.7×10^{-4}
**Male proband-female sibling pairs**	**Coding variants**	1.02	0.20	1.03	0.14
	**PTVs**	1.14	0.12	1.14	0.12
**Male proband-male sibling pairs**	**Coding variants**	1.01	0.67	1.01	0.67
	**PTVs**	1.27	4.8×10^{-3}	1.27	7.2×10^{-3}

Table S3 Number of samples from male proband-female sibling pairs, with matching or mismatching sequencing lane information, in each of the sequencing phases

	**Pilot**	**Phase1**	**Phase2**	**Phase3-1**	**Phase3-2**
**Matching lanes**	4	40	516	384	24
**Mismatching lanes**	32	404	10	6	154
